# Characterization of Intestinal Mycobiome in Surgical Resections from Inflammatory Bowel Disease Patients: A Deeper Analysis in Complicated Crohn’s Disease Phenotypes

**DOI:** 10.1093/ibd/izaf178

**Published:** 2025-10-30

**Authors:** Andrea Cejudo-Garcés, Miguel Carda-Diéguez, Francisco Navarro-Vicente, Sara Calatayud, Dolores Ortiz-Masiá, Álex Mira, María Dolores Barrachina, Jesús Cosín-Roger

**Affiliations:** Departamento de Farmacología, Facultad de Medicina, Universidad de Valencia, Valencia, Spain; Genomics and Health Department, FISABIO Foundation, Valencia, Spain; Departamento de Cirugía General y del Aparato Digestivo, Hospital de Manises, Valencia, Spain; Departamento de Farmacología, Facultad de Medicina, Universidad de Valencia, Valencia, Spain; CIBEREHD (Centro de Investigaciones en Red Enfermedad Hepática y Digestiva), Madrid, Spain; CIBEREHD (Centro de Investigaciones en Red Enfermedad Hepática y Digestiva), Madrid, Spain; Departamento de Medicina, Facultad de Medicina, Universidad de Valencia, Valencia, Spain; Genomics and Health Department, FISABIO Foundation, Valencia, Spain; CIBER Center for Epidemiology and Public Health, Madrid, Spain; Departamento de Farmacología, Facultad de Medicina, Universidad de Valencia, Valencia, Spain; CIBEREHD (Centro de Investigaciones en Red Enfermedad Hepática y Digestiva), Madrid, Spain; Departamento de Farmacología, Facultad de Medicina, Universidad de Valencia, Valencia, Spain; CIBEREHD (Centro de Investigaciones en Red Enfermedad Hepática y Digestiva), Madrid, Spain

**Keywords:** mycobiome, Crohn’s disease, ulcerative colitis, intestinal fibrosis

## Abstract

**Background:**

Inflammatory bowel disease (IBD), which encompasses ulcerative colitis (UC) and Crohn’s disease (CD), is a chronic condition characterized by recurrent intestinal inflammation and complications. Despite extensive research on bacterial dysbiosis in IBD, the role of the gut mycobiome remains largely unexplored, particularly in surgical tissue specimens.

**Methods:**

In this study, we performed a comprehensive analysis of the intestinal fungal communities in surgical resections obtained from 20 patients with UC and 30 patients with CD, with non-IBD resections serving as controls. Fungal DNA was extracted and the internal transcribed spacer (ITS) region was amplified and sequenced using high-throughput Illumina technology. RNA from surgical resections from both non-IBD and IBD patients was obtained and the expression of pro-inflammatory and profibrotic genes was analyzed by real-time quantitative polymerase chain reaction.

**Results:**

Bioinformatic analysis revealed modest changes in fungal diversity in UC resections compared with those from controls. However, CD specimens exhibited significant alterations in mycobiome composition, including an increased abundance of *Malassezia*, specifically *Malassezia globose*, alongside a reduction in *Yarrowia lipolytica*. Moreover, stratification of CD into complicated phenotypes (B2 stricturing vs B3 penetrating) identified distinct fungal signatures capable of discriminating between these clinical phenotypes. Correlation analyses revealed a direct association between the mycobiome and intestinal inflammation and fibrosis, in parallel with several interactions between fungal and bacterial species, further reporting interkingdom interactions between both microbial communities.

**Conclusions:**

These results underscore the potential of fungal biomarkers in elucidating IBD pathogenesis and its associated complications, which opens up promising avenues for targeted therapeutic strategies.

Key Messages *What is already known?*

Inflammatory bowel disease (IBD) patients exhibit mycobial dysbiosis, which points to the mycobiome as an important protagonist in IBD pathogenesis.

*What is new here?*

Intestinal surgical resections from IBD patients present a specific mycobial signature that also correlates with the intestinal bacteriome and the expression of pro-inflammatory and profibrotic genes. Moreover, complicated CD phenotypes exhibit significant differences in fungal species; specifically, *Dothideomycetes unclassified* and *Cladosporium unclassified* can discriminate between these phenotypes.

*How can this study help patient care?*

A more comprehensive characterization of the intestinal mycobiome could provide deeper insights into the pathogenic mechanisms of Crohn’s disease, support the prediction of its progression toward complicated phenotypes contributing to a better disease stratification and potentially guide the development of mycobiome-based pharmacological therapies, including the use of health-associated yeast probiotics.

## Introduction

Inflammatory bowel disease (IBD), which includes ulcerative colitis (UC) and Crohn’s disease (CD), is a chronic gastrointestinal disorder characterized by alternating periods of relapse and remission. Due to the lack of a pharmacological treatment that induces a complete remission of these diseases, IBD patients suffer these pathologies throughout their lives, which leads to the development of severe complications. In the case of CD, 2 phenotypes have been described depending on the developed complications: the B2 or stricturing phenotype, characterized by fibrosis and luminal narrowing, and the B3 or penetrating phenotype, characterized by the presence of fistulas and/or intra-abdominal abscesses.^1^^,^^2^ The etiology of IBD and its complications is still unclear, although it is assumed that a microbial alteration and an exacerbated activation of the immune system trigger the perpetuation of intestinal inflammation in genetically susceptible individuals.^3^

The gut microbiota is constituted by bacteria, archaea, virus (including phages), yeast, and fungi; and all these microorganisms interact with host cells regulating crucial biological functions such as the activation of immune response, the regulation of energy metabolism or glucose and lipid homeostasis, among others.^4^^,^^5^ While most previous research has focused specifically on the characterization of intestinal bacteria in IBD patients, fungal composition (mycobiome) has come under analysis only in recent years.

The gut mycobiome has been neglected and underestimated for so long probably due to the fact that it represents only 0.01%–0.1% of the overall intestinal microbial diversity. Nevertheless, it is important to consider that, in terms of size, fungi occupy a much larger area due to their big size and directly interact with several enteric bacterial pathogens that trigger both synergistic and antagonistic effects during the pathology’s progression.^6^ The intestinal mycobiome has been classified in resident and nonresident species. Whereas resident species, such as Ascomycota, Basidiomycota, and Zygomycota, can grow in the intestinal microenvironment; nonresident species, including Saccharomyces, Aspergillus, and Penicillium, among others, appear in the intestine due to an external insult, such as environmental factors or short-term dietary exposures.^7^^,^^8^ The gut mycobiome in IBD patients has been characterized in recent years and alterations in the *Basidiomycota/Ascomycota* ratio,^9^^,^^10^ reduced levels of *Saccharomyces*^11^^,^^12^ and increased levels of *Candida*^13^ have been reported in IL-6samples from these patients. Fungi composition has also been associated with different clinical parameters and outcomes in IBD patients, which highlights the importance of the mycobiome in IBD pathogenesis. Nevertheless, it is important to consider that these associations might be circumstantial instead of causative. In fact, levels of *Candida* have been associated with the clinical response to infliximab^14^ and with the efficacy of IL-6microbiota transplantation in patients with UC.^15^ Remarkably, most previous studies have been performed mainly in stool samples, and so very little is known about the fungal composition of intestinal tissue. In addition, the specific mycobial characterization of CD- complicated phenotypes has not yet been assessed, and, to our knowledge, only one study has specifically analyzed CD-associated phenotypes (only B1 and B2 phenotypes were compared).^10^

Therefore, in the present study we have characterized for the first time the mycobiome composition of intestinal surgical resections from both patients with UC and patients with complicated-CD. Here, we report differences in tissular fungal composition in both patients with UC and CD compared with healthy colons and ilea from non-IBD patients, respectively. In addition, we also describe a specific fungal signature that varied according to the complicated-CD phenotype.

## Materials and Methods

### Patients

Intestinal surgical resections of damaged tissue were obtained from UC (n = 20) and CD (n = 30) patients with severe refractory disease state who underwent surgery. The non-damaged mucosa of colonic (n = 18) and ileal (n = 12) resections from patients with colorectal cancer were used as controls of UC and CD, respectively. The Institutional Review Board of the Hospital of Manises (Valencia, Spain) approved the study with the number of code 2021-545-1. Written informed consent from all patients participating in this study was obtained. Table 1 provides the clinical information of our patients, including age, gender, classification, location and pharmacological treatment. None of the patients included in this study received any antibiotic before the surgery.

**Table 1.** Patient characteristics at the time of surgery.

	UC-Control	CD-Control	Patients with UC	Patients with CD
**Number of patients**	18	12	20	30
**Age median [interval]**	47 (28–85)	66 (24–89)	48,5 (17–69)	39,5 (18–77)
**Male/Female**	5/7	9/3	11/8	18/12
**Location**				
**Colon**	18		20	2
**Ileum**		12		28
**Classification**				
**A1**				
**A2**				16
**A3**				14
**L1**				25
**L2**				1
**L3**				4
**L4**				
**B1**				
**B2**				19
**B3**				11
**Pancolitis**			7	
**Distal colitis**			5	
**Proctosigmoiditis**			8	
**Treatment**				
**Infliximab**			1	2
**Azathioprine**				7
**Adalimumab**				1
**Azathioprine + Adalimumab**				1
**Azathioprine + Vedolimumab**				1
**Azathioprine + Corticoids + Anti-inflammatory Drugs**			3	
**Azathioprine + Corticoids**			1	3
**Azathioprine + Ustekinumab**				1
**Ustekinumab**			4	5
**Ustekinumab + Corticoids**				3
**Corticoids**			2	2
**Corticoids + Anti-inflammatory Drugs**			2	3
**Mesalazine**			5	
**Mesalazine + Corticoids**			1	
**Budesonide + Cortocoids**			1	

### DNA extraction, fungal load estimation and sequencing of the ITS fungal region

DNA extraction was performed as previously described, but including zymolase during the lysis step, together with lysozyme, lysostaphine and mutanolysin.^16^ Additionally, this mix was incubated for 15 min at 65°C with 20 ul of 1% Glucanex in PBS for additional lysis of fungal β-glucans. The MagNa Pure LC DNA Isolation kit II in a MagNa Pure Robot (Roche) was used for automatic DNA extraction following the protocol recommended by the manufacturer. DNA was quantified using the Quant-iT™ PicoGreen dsDNA Assay Kit and a Qubit™ 3 Fluorometer (ThermoScientific).

Fungal load was performed by qPCR in duplicates using fungal-specific primers targeted to the conserved ITS1–5.8S rRNA region, following the method by Boix-Amoros et al..^17^ In short, reaction mixtures were 20 μl in volume and were composed of 10 μl of KAPA Sybr Fast qPCR Kit (KAPA Biosistems), 0.4 μl of each primer at 10 μM concentration and 2 μl of template DNA. The fungal concentration in each sample was calculated by comparison with the Ct values obtained from a standard curve, generated with serial ten-fold dilutions of DNA extracted from 2 × 10^8^ fungal cells (as estimated by CFU counts) from pure cultures from different fungal species (*Candida albicans*, *Aspergillus niger*, *Sacharomyces boulardii*, *Thrichosporon cutaneum* and *Meyerozyme guilliermondii*), that were pooled to create a single standard curve. Bacterial load data for the same samples were retrieved from Bauset et al..^16^

For ITS Illumina sequencing, the hypervariable region ITS1 of the ITS gene was amplified using universal primers (forward ITS5 5′-GGAAGTAAAAGTCGTAACAAGG-3′ and reverse ITS2 5’-CTRYGTTCTTCATCGDT-3′).^18^ A total of 35 cycles (including the barcode indexing step) was performed for all samples. The Metagenomic Sequencing Library Preparation Illumina protocol (Part #15044223 Rev. A) was used for library preparation. Sequencing was performed at FISABIO Institute (Valencia, Spain) using 2 × 300 bp paired-end sequencing with an Illumina NextSeq instrument. Sequencing data have been publicly submitted to the SRA database (Bioproject: PRJNA1284784).

### Bioinformatic analysis of sequencing data

The software Dada2 v1.20 was used to filter, end-trim, denoise and merge paired reads^19^ using default parameters. Adapters and primers were first filtered out from the sequence reads and then end-trimmed in 10 bp windows with quality values <35 and absence of Ns. The remaining reads were merged, clustered and cleaned for host and chimeric reads and finally assigned a taxon at the genus and species level using the SILVA non-­redundant database v138.1.^20^

The R language was used for statistical computing to perform downstream analyses. For multivariant analysis, an Adonis test (permutational multivariate analysis of variance using distance matrices), provided by the Vegan library of R, was used to compare groups. Considering the minimum number of reads in a sample, rarefaction curves, richness and diversity indices were performed with 1000 sequences per sample. Nonparametric Wilcoxon tests (“wilcox.test” function of stats library of R) were performed to observe differences in the diversity indices between groups. To visualize differences among groups, constrained correspondence analysis (CCA) was carried out with the Vegan library. Additionally, to compare species and genera proportions, the abundance of taxons was transformed and compared using ANCOMBC2 approach. Adjusted *P* values were obtained by the FDR method.

### RNA extraction

Total RNA from human surgical intestinal resections was isolated using TRI Reagent Solution (Invitrogen, Thermo Fisher Scientific). Tissue samples were homogenized using Cryolys evolution system in 800 μL of trizol, with 3-4 metal beads. Homogenization was performed in 3 50-second cycles, each followed by 20-second pause. The homogenates were then centrifuged at 15 000 *g* for 15 minutes at 4°C. Subsequently, 200 μL of chloroform were added to the supernatant, followed by vigorous vortexing. Samples were incubated on ice for 15 minutes and centrifuged again at 15 000 *g* for 15 minutes at 4°C.

The colorless aqueous (upper) phase was carefully transferred to new tubes in which RNA was precipitated by adding 500 μL of isopropanol and was incubated overnight at −20°C. The following day, RNA was pelleted by centrifugation at 15 000 × g for 20 minutes at 4°C, washed with 1 mL of 70% ethanol, centrifuged again, and finally resuspended in 30 μL of RNAse-free water.

### Complementary DNA synthesis by reverse-transcription

Complementary DNA (cDNA) was synthesized by reverse transcription using PrimeScript RT reagent Kit (Perfect Real Time; TaKaRa Bio, Otsu, Japan), according to the manufacturer’s instructions. For each reaction, 1 μg of total RNA was used in a final volume of 20 μL, containing 4 μL of 5x PrimeScript Buffer, 1 μL PrimeScript RT Enzyme Mix I, 1 μL of 25-pmol Oligo(dT) Primer and 1 μL of 50-pmol Random Hexamers. Reverse transcription was performed in a GeneAmp PCR System 2400 thermal cycler (PekinElmer Inc, Waltham, MA, USA) under the following conditions: 15 minutes at 37°C, 5 seconds at 85°C, followed by storage at 4°C until samples were stored at −20°C.

### Real-time quantitative PCR

Real-time quantitative PCR (RT-qPCR) was performed using TB Green Premix Ex Taq (Tli RNAseH Plus; TaKaRa Bio) which contains TaKaRa Ex Taq HS, a dNTP mix, Mg^2+^, Tli RNase H, and SYBR Green I—a fluorophore that emits fluorescence upon binding to double-stranded DNA, enabling real-time detection and quantification of amplified products.

Each 10-µL reaction mixture contained 1 µL of cDNA, 5 µL of TB Green Premix Ex Taq, 0.3 µL each of forward and reverse primers, and RNase-free H2O to reach the final volume. RT-qPCR was carried out in a QuantStudio Real-Time PCR System (appliedbiosystems; Thermo Fisher Scientific) under the following conditions: initial denaturation at 95°C for 30 seconds, followed by 50 cycles of 95°C for 5 seconds and 60°C for 20 seconds, and a final dissociation protocol consisting of 95°C for 1 seconds, 65°C for 15 seconds, 95°C for 1 seconds, and 40°C for 30 seconds.

All reactions were performed in duplicate, and a no-template control (NTC) using RNase-free water instead of cDNA was included in each run. Primer sequences for human targets are listed in Table 2. Primer specificity was confirmed by melting curve analysis and electrophoresis on a 2% agarose gel containing Serva DNA Stain G (Serva) in 1X TAE buffer (20-mM Tris pH 7.8, 0.5-mM Ethylenediaminetetraacetic acid (EDTA), 10 mM sodium acetate). DNA bands were visualized using the AMERSHAM ImageQuant 800 system (GE Life Sciences; Cornellà de Llobregat, Spain). Gene expression levels were reported as ΔCT values, using *ACTB* as the housekeeping gene.

**Table 2.** Sequences of human primers used in RT-qPCR.

Gene	Sense (5′-3′)	Antisense (5′-3′)	Size (bp)
*ACTB*	GGACTTCGAGCAAGAGATGG	AGCACTGTGTTGGCGTACAG	57
*COL1A1*	AGCACTGTGTTGGCGTACAG	CCGTTCTGTACGCAGGTGAT	252
*COL3A1*	CGCCCTCCTAATGGTCAAGG	TTCTGAGGACCAGTAGGGCA	161
*COL4A1*	CCGGATCACATTGACATGAAACC	TGGAAACCAGTCCATGCTCG	236
*IL-1B*	GCTCGCCAGTGAAATGATGG	TCGTGCACATAAGCCTCGTT	330
*IL-6*	CTCAATATTAGAGTCTCAACCCCCA	GAGAAGGCAACTGGACCGAA	143
*IL-8*	CCACCGGAGCACTCCATAAG	GATGGTTCCTTCCGGTGGTT	97
*IL-10*	CCTGCCTAACATGCTTCGAG	TCTTGGTTCTCAGCTTGGGG	198
*IL-17*	AACGATCCACCTCACCTTG	TAGTCCACGTTCCCATCAGC	223

### Correlation analysis

Bacterial composition data corresponding to the same samples were obtained from our previous publication^16^ with the accession number PRJNA859102. Results derived from the mycobial sequencing were correlated with the 16S-basaed microbiota sequencing, the clinical parameters age at the moment of surgery and sex, and ΔCT values of the genes quantified by qPCR using the Spearman correlation, since it detects more complicated relationships that might otherwise go undetected when using other metrics, such as the Pearson correlation. Values of Spearman correlation coefficients were represented in a heatmap using GraphPad software. The *P* values of each correlation were also represented in a separated heatmap.

### Statistical analysis

Data were expressed as mean ± SEM and compared using a *t* test for comparisons between 2 groups and 1-way analysis of variance (ANOVA) with Tukey post hoc correction or ­Kruskal-Wallis with Dunn post hoc correction where appropriate for multiple comparisons. A *P* value <.05 was considered to be statistically significant.

## Results

### Clinical characteristics of patients included in the study

A total of 80 intestinal surgical resections from non-IBD (*n* = 30) and IBD patients (*n* = 50) were included in this study. Specifically, 20 patients with UC and 30 with CD who had undergone surgery were recruited from the Hospital of Manises. Given the different localization of UC and CD lesions, a healthy tissue from colon (n = 18) or ileum (n = 12) from 30 patients with colon carcinoma requiring surgery were also recruited from Hospital of Manises in order to use them as controls for patients with UC and CD respectively. The mean ages of the 4 groups were as follows: patients ages 53.3 years ± 6.2 for control-UC, 60.6 ± 5.7 for control CD, 46.4 ± 4.6 for UC and 42.5 ± 3.0 for CD. There were no statistical differences in the male/female proportion among the groups in this study. The clinical information for both patients with UC and CD regarding location, classification, and pharmacological treatment is presented in Table 1.

### Fungal mycobiome load and diversity in surgical resections from IBD patients

**Figure 1.**
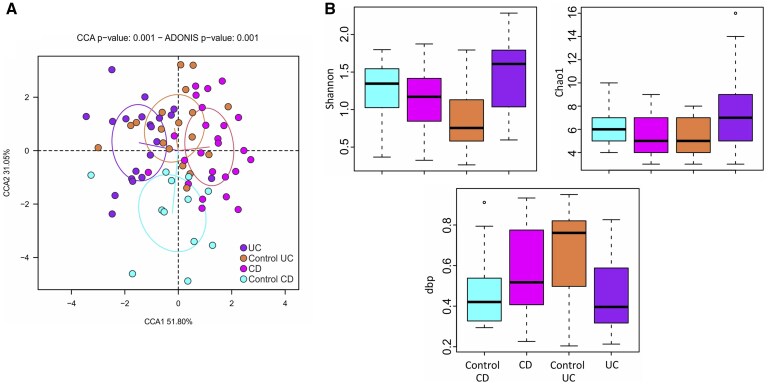
Characterization of intestinal mycobiome in intestinal surgical resections from IBD patients. (A) The intestinal mycobiome composition was compared between groups in a Canonical Correspondence Analysis plot using ADONIS test. (B) Graphs show mycobial diversity (Shanon), mycobial richness index (Chao1) and the dominance index (dbp) in the 4 groups included in the study: control-UC (*n *= 18), control CD (*n* = 12), patients with UC (*n* = 20), and patients with CD (*n* = 30).

In order to characterize the intestinal mycobiome in surgical resections from patients with non-IBD and IBD, DNA was amplified with universal primers for the ITS hypervariable region. First, as shown in Figure 1(A), the 4 groups included in the study were compared by a Canonical Correspondence Analysis and control-UC and control CD clustered separately (ADONIS, *P = .*013). Surgical resections from patients with UC partially overlapped with control-UC samples (ADONIS *P = .*16), whereas intestinal samples from patients with CD grouped separately from control CD samples (ADONIS *P = .*006).

Analysis of diversity (Shannon), richness (Chao1) and dominance (dbp) indices of surgical resections from non-IBD patients exhibited no significant differences between control-UC and control CD samples. Similarly, no significant differences in the diversity indices were detected between control-UC and UC nor control CD and patients with CD. Only patients with UC showed a tendency for an increase in Shannon and Chao1 indices in parallel with a reduction in the dpb index with respect to control-UC samples; whereas CD samples showed a slightly higher dpb index compared with control CD surgical resections (Figure 1(B)).

In addition to the fungal composition, the DNA from the same samples was used to estimate fungal load by qPCR. An average of 64.3 ± 17.6 CFUs per gr of tissue was obtained. This was 1406 times lower than the estimated bacterial load in the same samples, as assessed by qPCR with bacterial-specific primers.

### Characterization of intestinal mycobiome in surgical resections from IBD patients

**Figure 2.**
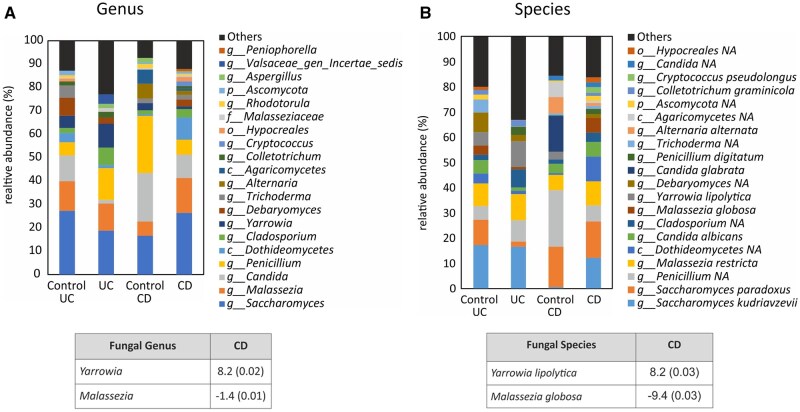
Intestinal mycobiome composition at genus and species level in intestinal surgical resections from IBD patients. Graphs show mycobial composition at genus level (A) and species level (B) in the 4 groups of samples, determined by the sequencing of DNA after its amplification with universal primers for internal transcribed spacer (ITS) hypervariable region in intestinal surgical resections of control-UC (*n* = 18), control CD (*n* = 12), patients with UC (*n* = 20), and patients with CD (*n* = 30) The tables included in this figure show the genus and species with statistical significance indicating the log2FC and the *P* values in brackets.

Characterization of the mycobiome at genus level revealed differences in the relative abundance of the top 20 most abundant genera. Figure 2(A) shows the relative abundance of those most abundant genera in the 4 groups included in the study. Despite the different fungal signature, statistical significance was not reached in any of the genera when samples from patients with UC and control-UC samples were compared. Nevertheless, resections from patients with CD showed a significant increase in the genus *Malassezia* (log2FC = −1.4) and a significant reduction in the genus *Yarrowia* (log2FC = 8.2) compared to control CD samples.

Next, a more in-depth characterization of fungal composition at the species level was performed. In this case, Figure 2(B) indicates the relative abundance of the top 20 most abundant species in the samples analyzed. Whereas no significant differences were obtained in any species between UC and control-UC samples, some statistical differences were reported between CD and control CD samples. In fact, a significant increase in the *Malassezia globose* (log2FC = −9.4, *P = .*02) and a significant reduction in *Yarrowia lipolytica* (log2FC = 8.2, *P = .*02) were obtained in CD vs control CD resections. The donor-specific breakdown of fungal relative abundance at both genus and species level is included in Figure S1(A).

### Correlation between the mycobiome and intestinal microbiota in surgical resections from IBD patients

**Figure 3.**
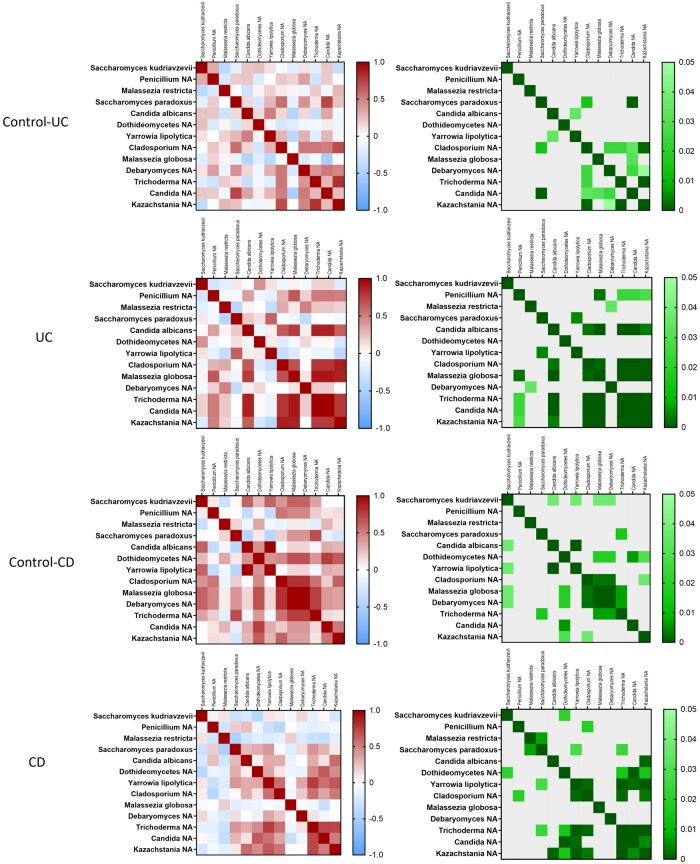
Fungal correlations in intestinal surgical resections from IBD patients. The heatmaps on the left show the Spearman correlation coefficient of the correlation between the fungal species in intestinal surgical resections of control-UC (*n* = 18), control CD (*n* = 12), patients with UC (*n* = 20), and patients with CD (*n* = 30) separately. The heatmaps on the right show the *P* value of each correlation.

Once we had characterized the intestinal mycobiome in surgical resections from both patients with UC and CD, we sought to analyze the relationship between the abundance of fungal species in both diseases in order to better understand their interactions. As shown in Figure 3, the correlations of the 2 control groups, control-UC and control CD, revealed a specific profile depending on the colonic or ileal tissue. For instance, in control-UC samples, the strongest positive and significant correlations were obtained between *Saccharomyces paradoxus* and *Candida unclassified*, and *Kazachstania unclassified* between *Cladosporium unclassified* and *Trichoderma unclassified*. In control CD samples, the strongest positive and significant correlations were detected between *Cladosporium unclassified, Malassezia globose*, *Debaryomyces unclassified*, and *Trichoderma unclassified*. On the other hand, in surgical samples from patients with UC, most of the correlations were positive, whereas no negative correlation reached statistical significance. The strongest and significant positive correlations found in UC samples were detected between *Candida albicans*, *Cladosporium unclassified*, *Malassezia globose*, *Trichoderma unclassified*, *Candida unclassified*, and *Kazachstania unclassified*. In contrast, patients with CD showed a different pattern of correlations. Specifically, negative and significant correlations were found between *Cladosporium unclassified* and *Penicillium unclassified, Saccharomyces kudriavzevii* and *Dothideomycetes unclassified*. The strongest and most significant positive correlations were found between *Yarrowia lipolytica, Dothideomycetes unclassified*, *Trichoderma unclassified*, *Candida unclassified*, and *Kazachstania unclassified*.

**Figure 4.**
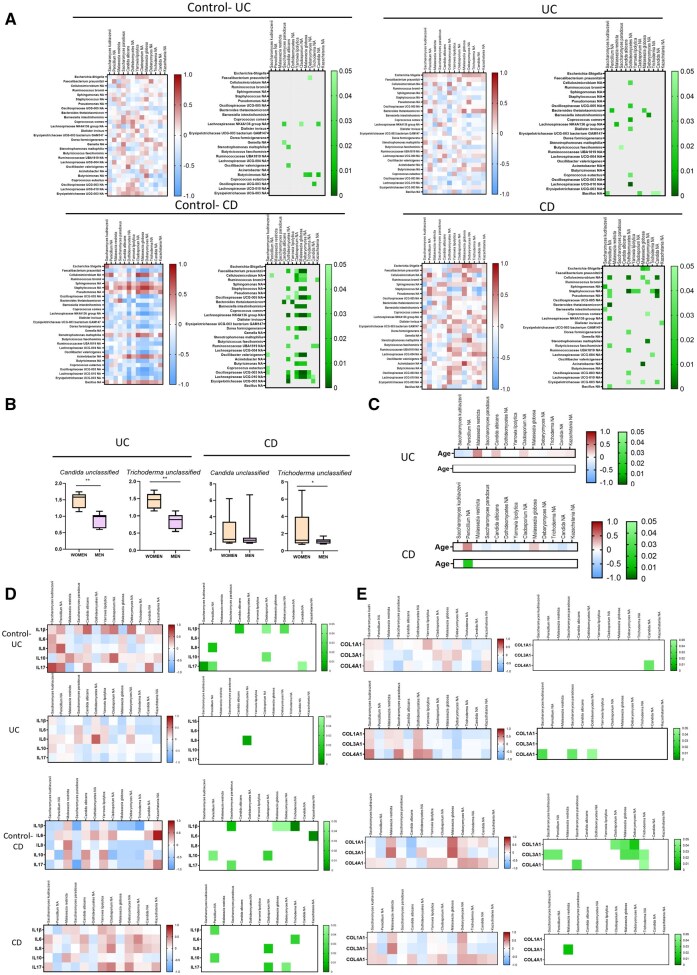
Fungal species correlate with bacterial species, the sex, the age at the moment of surgery, and the expression of pro-inflammatory and profibrotic genes in intestinal surgical resections from IBD patients. (A) The heatmaps with blue and red colors show the Spearman correlation coefficient of the correlation between the fungal species and bacterial species in intestinal surgical resections of control-UC, control CD, patients with UC, and patients with CD separately. In addition, the heatmaps with the green color show the *P* value of each correlation. (B) Graphs show the relative abundance of *Candida unclassified* and *Trichoderma unclassified* in surgical resections from both patients with UC and CD depending on the sex. (C) Heatmaps showing the Spearman correlation coefficient of the correlation between the relative abundance of fungal species and the age at the moment of surgery and the *P* value of each correlation in both patients with UC and CD. (D) Heatmaps showing the Spearman correlation coefficient of the correlation between the –ΔCts values of pro-inflammatory cytokines and the relative abundance of fungal species and the *P* value of each correlation in each group of samples. (E) Heatmaps showing the Spearman correlation coefficient of the correlation between the –ΔCts values of profibrotic genes and the relative abundance of fungal species and the *P* value of each correlation in each group of samples.

To explore potential interkingdom interactions between intestinal bacteria and mycobiome, we correlated the proportion of fungal species and the intestinal microbiota we recently reported in the same surgical resections from both patients with UC and CD.^16^ As shown in Figure 4, few significant correlations were detected between the bacteriome and mycobiome in intestinal resections from control-UC and from patients with UC and CD, whereas a high number of significant correlations were obtained in control CD surgical resections. In fact, significant and negative correlations were detected between the fungal species *Cladosporium unclassified, Malassezia globose*, and *Debaryomyces unclassified* and the bacterial species *Faecalobacterium prausnitzii, Ruminococcus bromii,* an unclassified *Staphylococcus,* an unclassified *Oscillospiraceae UCG-005, Barnesiella intestinihominis, Lachnospiraceae NK4A136, Dorea formicigenerans, Oscillibacter valericigenes,* an unclassified *Butyricimonas,* an unclassified *Oscillospiraceae UCG-003* and an unclassified *Lachnospiraceae UCG-010*. In patients with UC, *Dothideomycetes unclassified* was the fungi with the most significant correlations with different bacteria. Specifically, it exhibited a strong positive correlation with *Cellulosimicrobium unclassified* in parallel with negative correlations with *Oscillospiraceae UCG-005, Coprococcus comes, Coprococcus eutactus, Dialister invisus*, and *Lachnospiraceae UCG-010*. On the other hand, in patients with CD, most of the negative and significant correlations found in control CD samples were lost, while new significant correlations appeared in these pathological samples. Specifically in these patients, *Dothideomycetes unclassified* positively correlated with *Dorea Formicigenerans, Ruminococcaceae UBA 1819, Oscillospiraceae UCG-003* and *Erysipelotrichaceae UCG-003*, whereas it negatively correlated with *Cellulosimicrobium unclassified* and *Staphylococcus unclassified*. In addition, *Malassezia globose, Penicillium unclassified, Debaryomyces unclassified*, and *Candida unclassified* also showed positive correlations with different bacterial species. *Penicillium unclassified* positively correlated with *Cellulosimicrobium unclassified, Staphylococcus unclassified*, and *Pseudomonas unclassified*, while *Candida unclassified* correlated in the opposite way. Moreover, *Penicillium unclassified* negatively correlated with *Bacteroides thetaiotaomicron, Lachnospiraceae NK4A136, Butyricicoccus faecihominis*, and *Bacillus unclassified*.

### Intestinal mycobiome correlates with the clinical parameters sex and age at the surgery and with the expression of pro-inflammatory and profibrotic genes

Next, based on the microbial differences depending on both the sex and age described in different pathologies including IBD,^21^^,^^22^ we analyzed whether clinical parameters influence the intestinal mycobiome. As shown in Figure 4(B), in patients with UC, of all fungi analyzed, only *Candida unclassified* and *Trichoderma unclassified* were significantly reduced in men compared with women. In our patients with CD, only *Trichoderma unclassified* was significantly reduced in male vs female samples. In relation to the age at the moment of surgery, no significant correlation between this parameter and the fungal species was detected, except *Penicillium unclassified*, which showed a positive and significant correlation with age specifically in patients with CD (Figure 4(C)).

In addition, in order to deeper study whether intestinal mycobiome is associated with intestinal inflammation, we quantified the gene expression of several pro-inflammatory cytokines in the same surgical resections in which we have characterized the intestinal mycobiome, and we have correlated the expression of those genes with the fungal levels. As shown in Figure 4(D), the correlation profiles were different depending on the group of samples analyzed. In control-UC samples, positive and significant correlations were obtained between *IL-1β* and *Candida albicans*, *Cladosporium NA* and *Debaryomyces NA*, between *IL-8* and *Penicillium NA* and between *IL-17* and *Saccharomyces kudriavzevii*, *Penicillium NA* and *Candida NA*; whereas UC samples only exhibited a positive and significant correlation between *IL-8* and *Dothideomycetes NA*. On the other hand, control CD samples showed negative and significant correlations between *IL-1β* and *Saccharomyces parodoxus*, *Malassezia globose*, *Debaryomyces NA*, and *Trichoderma NA*, in parallel with a strong positive and significant correlation between *IL-6* and *Kazachstania NA*. However, CD samples exhibited a different pattern of correlation showing a positive and significant correlation between *Cladosporium NA* and *IL-8* and *IL-17*, *Trichoderma NA* and *IL-6* and *Debaryomyces NA* and *IL-17*.

Growing evidence indicates that intestinal microbiota exerts an important role in the development of intestinal fibrosis.^23^ Hence, we have also correlated the expression of the profibrotic genes *COL1A1*, *COL3A1* and *COL4A1* with the levels of the fungal species of the same intestinal resections. As shown in Figure 4(E), we obtained fewer significant correlations compared with the previous correlations with pro-inflammatory cytokines. In this case, in control-UC samples we only detected a negative and significant correlation between *Candida NA* and *COL4A1*, while UC samples showed positive and significant correlations between *COL4A1* and *Saccharomyces kudriavzevii*, *Saccharomyces parodoxus*, and *Dothideomycetes NA*. On the other hand, control CD samples showed a high number of positive and significant correlations between *COL1A1* and *Cladosporium NA*, *Malassezia globose* and *Debaryomyces NA* and between *COL3A1* and *Saccharomyces kudriavzevii*, *Penicillium NA*, *Malassezia globose*, *Debarymyces NA*, and *Trichoderma NA*. Nevertheless, CD resections only showed a positive correlation between *Malassezia restricta* and *COL3A1*.

### Phenotypes of complicated-CD exhibit a different mycobiome

**Figure 5.**
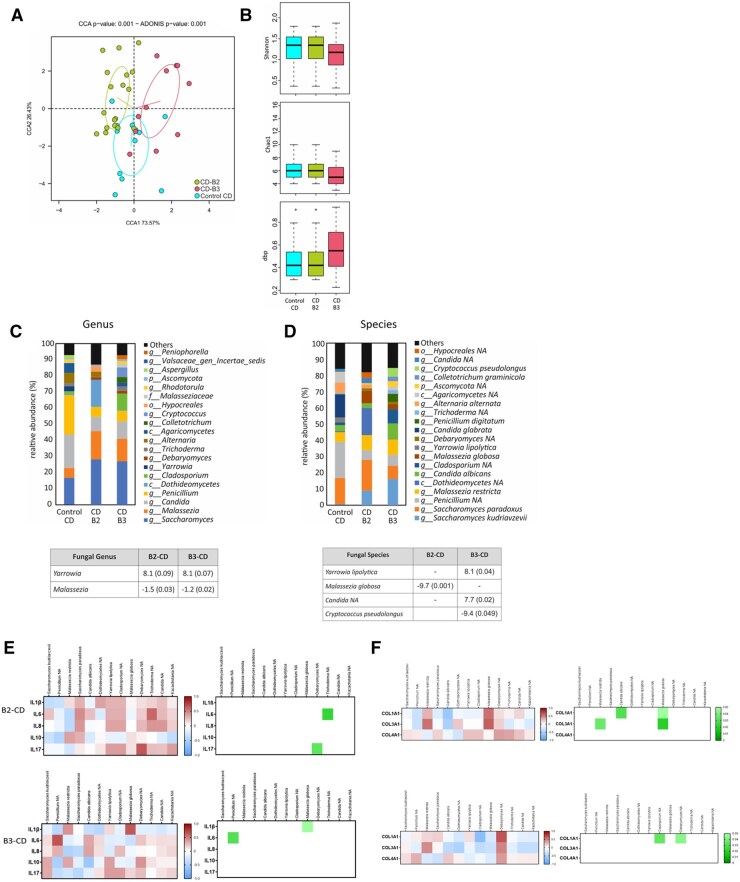
Characterization of intestinal mycobiome in intestinal surgical resections from complicated-CD phenotypes. (A) The intestinal mycobiome composition was compared between groups in a Canonical Correspondence Analysis plot using ADONIS test. (B) Graphs show mycobial diversity (Shanon), mycobial richness index (Chao1), and the dominance index (dbp) in the 3 groups included: control CD (*n* = 12), B2-CD (*n* = 19), and B3-CD (*n* = 11) patients. (C, D) Graphs show mycobial composition at genus level (C) and species level (D) in the 3 groups of samples, determined by the sequencing of DNA after its amplification with universal primers for ITS hypervariable region in intestinal surgical resections of control CD, patient with B2-CD, and patients with B3-CD. The tables included in this figure show the genus and species with statistical significance indicating the log2FC and the *P* values in brackets. (E) Heatmaps showing the Spearman correlation coefficient of the correlation between the −ΔCts values of pro-inflammatory cytokines and the relative abundance of fungal species and the P value of each correlation in B2-CD and B3-CD samples. (F) Heatmaps showing the Spearman correlation coefficient of the correlation between the −ΔCts values of profibrotic genes and the relative abundance of fungal species and the *P* value of each correlation in B2-CD and B3-CD surgical resections.

Once the mycobiome had been characterized in both patients with UC and CD, we decided to analyze whether the different complicated CD-phenotypes are associated with a specific fungal composition. For that aim, we divided patients with CD into B2 or B3 depending on the complicated phenotype developed. As shown in Figure 5(A), B2-CD and B3-CD samples grouped separately using a multivariable approach with an ADONIS *P* value of .001. However, no statistical differences were obtained in the diversity, richness, or dominance indices between B2-CD and B3-CD surgical resections (Figure 5(B)).

Additionally, we analyzed differences in mycobiome composition between B2-CD and B3-CD complicated phenotypes. ­Figure 5(C) shows the 20 most abundant genus in each of the groups of samples analyzed. At the genus level, significant differences were observed between B2-CD and B3-CD samples (Figure 5(C)). Of interest, in both phenotypes there was an increase in the abundance of *Malassezia* when compared with control CD, in parallel with a reduction in the abundance of *Yarrowia*.

At species level, significant differences were detected as shown in Figure 5(D). In fact, differences in the abundance of *Malassezia globose* were statistically significant; patients with B2 CD specifically showed a significant increase in this species compared to control CD samples, whereas B3-CD samples exhibited a slight reduction vs control CD samples. Besides *Malassezia globose*, other species varied significantly in B3-CD vs control CD. In particular, *Candida unclassified* and *Yarrowia lipolytica* were lower in B3-CD than in control CD resections. In addition, in order to determine specific fungal signatures for the 2 complicated phenotypes of patients with CD, the mycobiome composition of B2-CD and B3-CD samples were compared. *Cryptococcus pseudolongus* and *Cladosporium unclassified* were significantly more abundant in B3-CD samples than in B2-CD samples, whereas *Dothideomycetes unclassified* was more abundant in B2-CD than in B3-CD samples. The donor-specific breakdown of fungal relative abundance at both genus and species level is included in Figure S1(B).

After characterizing the intestinal mycobiome specifically in complicated-CD’s phenotypes, we also correlated the fungal levels with the expression of several pro-inflammatory and profibrotic genes in order to deeper analyze the relationship between intestinal inflammation and fibrosis in these complicated CD-phenotypes with the fungal signature. As shown in Figure 5(E), we have only obtained in B2-CD samples 2 positive and significant correlations between *IL-6* and *Trichoderma NA* and *IL-17* and *Debaryomyces NA*; whereas in B3-CD samples, 2 strong positive and significant correlations were obtained between *IL-1β* and *Malassezia globose* and *IL-6* and *Penicillium NA*. Regarding the association with intestinal fibrosis in patients with B2 with CD, we detected positive and significant correlation between *Malassezia globose* and *COL1A1* and *COL3A1* and *Malassezia restricta* with *COL3A1*  Figure 5(F); while B3-CD samples exhibited a strong a positive correlation between *COL1A1* and *Debaryomyces NA*.

### Fungal mycobiome discriminates complicated-CD’s phenotypes

Finally, we sought to study whether fungal species were able to discriminate between the 2 complicated-CD phenotypes (B2 and B3). To do this, receiver operating characteristic (ROC) curves for the most abundant species were generated and the area under the curve (AUC), *P* value, and the 95% CI for each fungal species have been included in Table S1. Among all the fungal species, *Dothideomycetes unclassified* and *Cladosporium unclassified* showed statistically significant ROC curves with an AUC of 0.8232 and 0.7374, respectively. In addition, *Malassezia globose* almost reached statistical significance (*P = .*0876), with an AUC of 0.6919.

## Discussion

The study of the gut microbiome and its effect on gut disorders has been heavily biased towards the bacterial component. This is understandable due to the disproportionately larger number of bacterial than fungal cells in most human niches, in agreement with our data showing a 1000-fold lower number of fungi compared to bacteria. Although the low fungal density could imply a reduced biological significance in the gut ecosystem, several studies provide quantitative size fungal: bacterial ratios of 100x in volume.^24^^,^^25^ Additionally, the structural and metabolic influence of fungi should also be considered. For example, hyphae and large yeast cells act as a physical scaffold in polymicrobial biofilms, shaping bacterial community architecture and local oxygen, pH and nutrient gradients.^26^ Finally, other authors have reported that altering host sensing of commensal fungi (via attenuation of the Dectin-1 receptor) is sufficient to tip the balance from mild to severe colitis, even though the absolute fungal load remains orders of magnitude below the bacterial load.^27^ These data therefore suggest that function—and not cell count—may drive biological relevance. The present study describes, for the first time to our knowledge, the fungal composition of surgical resections from both patients with UC and CD and reveals a specific intestinal mycobiome in these patients. In addition, we have identified specific fungal signatures depending on the complicated CD-associated phenotypes.

In recent years, a mycobiome dysbiosis has been reported in IBD patients.^28^^,^^29^ While most studies have characterized the fungal composition of fecal samples,^30^^,^^31^ we report here for the first time a mycobiome dysbiosis specifically in surgical intestinal resections from IBD patients. In our cohort of patients, the surgical resections showed nonsignificant differences in Chao1, Shannon and dbp indices between pathological and nonpathological samples. In fact, we have observed only a slight increase in the Chao1 and Shannon indices in patients with UC compared with control-UC samples, suggesting that the richness and diversity is slightly increased in patients with UC. It is important to note that both an increase^32^ and decrease^9^ fungal diversity have been described in fecal samples from IBD patients. Moreover, the different human samples included in previous studies must be taken into consideration. Indeed, there is controversy surrounding reports of increased^32^ and decreased^33^^,^^34^ fungal diversity in intestinal biopsies from IBD patients. In addition, apart from the few studies using intestinal samples, to our knowledge only one has employed surgical resections, and which used intestinal biopsies as controls.^33^ Therefore, it is worth to highlight the use as controls of surgical resections from the same affected location (colon or ileum) and a reasonable number of samples from each group.

Characterization of the intestinal mycobiome in surgical colonic resections from patients with UC revealed nonsignificant differences at the genus and species levels compared with control-UC resections. This goes against previous research showing a mycobial dysbiosis specifically in fecal samples^35^ or intestinal biopsies^36^ of patients with UC vs non-IBD patients. Interestingly, it has recently been reported that the mycobiome is dynamic and associated with clinical activity in fecal samples from patients with UC,^37^ presenting changes after an intestinal resection associated with postoperative and disease outcomes.^38^ These recent and elegantly performed studies, together with our results, help to explain discrepant research and suggest that surgical resections obtained from patients with UC, when patients present the most severe state of the disease, do not exhibit mycobial dysbiosis. Nevertheless, 2 limitations of this study must be considered. Firstly, although we studied a considerable number of intestinal resections, and despite the difficulty of obtaining such samples, our results would need to be validated in another cohort of patients with UC. In addition, nondamaged colonic tissue from colon-carcinoma patients were included as control samples, since it is not possible to obtain healthy colonic surgical resections. In this way, further studies are needed to better characterize the intestinal mycobiome of patients with UC with a different control in order to confirm our findings. For instance, the nondamaged mucosa of the same patient might provide important information describing the intestinal mycobiome specifically in the healthy tissue of these patients.

In contrast, when compared with control CD samples, our patients with CD presented a significant increase in the genus *Malassezia*, specifically the species *Malassezia globose*, in parallel with a significant reduction in the genus *Yarrowia*, specifically the species *Yarrowia lipolytica*. In this case, our results are in line with several studies showing a significant increase in the *Malassezia* genus in intestinal biopsies,^39^^,^^40^ in fecal samples^30^ and even in mesenteric adipose tissue from patients with CD.^41^ The fact that the fungal alterations found in surgical resections have also been observed in both intestinal biopsies and fecal samples, suggest that tissular mycobial alterations are kept through the whole tissue and even in the stool, what makes these fungi interesting biomarkers. Our results not only expand on previous observations confirming an increased abundance of *Malassezia globose* species in the whole thickness of the intestinal layer, but also provide evidence for the first time of a significant reduction in the genus *Yarrowia*, specifically *Yarrowia lipoyitica*. This fungal species is considered part of the homeostatic intestinal mycobiota since it has been isolated from stool samples from healthy people;^42^ moreover, as far as we know, only one study has reported a negative correlation between the presence of the *Yarrowia* species in the intestine and the efficacy of IL-6microbiota transplantation.^43^ The fact that alterations in the abundance of *Yarrowia* have never been detected in IBD patients suggests that the reduction observed in our CD-resections point to a specific location of this fungus in the most superficial layers of the intestinal tissue, which are commonly lost in damaged tissue of patients with CD due to the transmural lesions. In this context, future in situ hybridization studies are vital in order to localize *Yarrowia* throughout the whole intestine. From an applied viewpoint, *Yarrowia* strains have emerged as promising candidates for yeast probiotics,^44^ and further experimental research should evaluate its potential antimicrobial and anti-inflammatory properties with potential benefits for IBD patients, specifically in those with complicated phenotypes. In patients with CD, it is important to note that there is a difference in the age between control CD and patients with CD which should be taken into account as a limitation of this study. As in the case of patients with UC, nondamaged mucosa of surgical resections from the same patients with CD could be important controls. Future studies analyzing specifically nondamaged and damaged mucosa of the same patients should be performed in order to better describe the intestinal mycobial alterations in both patients with UC and CD.

Our correlation analysis between fungal species revealed different interactions depending on the intestinal part and the pathology. While control-UC samples showed positive and significant correlations between *Kazachstania unclassified* and *Cladosporium unclassified* and *Trichoderma unclassified*; control CD samples exhibited a different pattern of correlations with a higher number of positive and significant associations. Regarding pathological tissues, in UC samples only strong and positive correlations were observed between some fungal species such as *Candida Albicans*, *Cladosporium unclassified, Malassezia globose, Trichoderma unclassified, Candida unclassified* and *Kazachstania unclassified*. These results, together with previous evidence reporting a pathogenic role of these fungal species,^45–47^ suggest that pathogenic fungal species promote the growth of other pathogenic species in these patients and probably exacerbate intestinal inflammation. On the other hand, patients with CD exhibited weaker positive correlations and few negative correlations between *Cladosporium unclassified* and *Penicillium unclassified, Saccharomyces kudriavzevii* and *Dothideomycetes unclassified*. The fact that fungal species correlated differently depending on the pathology implies the presence of another factors or protagonists, for instance, bacterial species, which modify these interactions.

There is growing evidence of interactions between bacterial and fungal intestinal species in an interkingdom where bacteria and fungi share similar niches and coexist, directly interacting amongst themselves.^24^^,^^48^^,^^49^ At this point, it is important to highlight that correlations between the bacteriome and mycobiome in the same patient have only recently been described by Wetzel et al in fecal samples,^38^ while no study has used intestinal surgical resections, as far as we know. While control-UC samples exhibited a reduced number of significant correlations, control CD samples showed an increased number of significant and negative correlations between the fungal species *Cladosporium unclassified, Malassezia globose*, and *Debaryomyces unclassified* and several bacterial species such as *Faecalobacterium prausnitzii, Ruminococcus bromii* and *Dorea formicigenerans,* among others. In our cohort of IBD patients, the fungal species most significantly correlated with bacterial species were *Dothideomycetes unclassified* in both patients with UC and CD and *Penicillium unclassified, Malassezia globose, Debaryomyces unclassified* and *Candida unclassified* only in patients with CD. These results strongly reinforce the idea of a connection between bacterial and fungal species in both pathologies and shed light on specific microbial connections which need to be further studied. Moreover, given the different location of affected tissue in both pathologies, the correlations between *Dothideomycetes* and several bacterial species detected in both patients with UC and CD suggest that these associations are related specifically to intestinal inflammation, regardless of the specific area of the intestine.

Sex-based differences in intestinal microbiota have been reported in different diseases, including IBD.^22^ Herein we demonstrate that, among all the fungal species detected in surgical resections from IBD patients, only *Trichoderma unclassified* and *Candida unclassified* are significantly reduced in men compared with women. In line with our results, higher levels of *Candida* species were described in infections of denture wearers that were female vs male.^50^ Besides sex, the human gut microbiota also evolves in the host during the whole lifetime, resulting in age-related microbial alterations.^51^ The association between the age at the surgery and the composition of colon- or ileum-associated mycobiome in our cohort of IBD patients revealed nonsignificant correlations between both parameters, except for *Penicillium*, which showed a significant and positive correlation with the age only in patients with CD. In accordance with our results, an age-dependent increase in *Penicillium* has been reported in fecal samples from healthy male human volunteers.^52^

Positive and significant correlations between the expression of pro-inflammatory and profibrotic genes with the relative abundance of fungal species pointed to a pathogenic effect of some mycobial species. In fact, *Dothideomycetes* exhibited a positive correlation with *IL-8* in UC samples, whereas *Cladosporium NA, Debaryomyces NA* and *Trichoderma NA* positively correlated with the expression of *IL-6*, *IL-8*, and *IL-17*. In line with our results, a pathogenic role of both *Cladosporium* and *Debaryomyces* has been reported. Indeed, the infection with *Cladosporium cladosporioides* increased the expression of pro-inflammatory cytokines including *IL-17* in the skin and the lung,^53^^,^^54^ whereas the infection with *Debaryomyces*, which is a potent trigger of Th17 response, impaired intestinal mucosal healing.^55^ Regarding the correlations with profibrotic genes, it is important to highlight that both *Malassezia globose* and *Malassezia restricta* showed the strongest and positive correlations with *COL1A1* and *COL3A1* suggesting a profibrotic role of these fungi. The pathogenic role of *Malassezia restricta* was reported by Limon and colleagues who demonstrated an exacerbation of acute murine dextran sulphate sodium (DSS)-induced colitis.^39^ Nevertheless, to our knowledge, this is the first study which associates these *Malassezia* species with intestinal fibrosis. The increased abundance of this fungi in patients with CD and their direct effect in regulating inflammation and, probably, intestinal fibrogenesis, point this fungal species as key protagonists in CD’s pathophysiology. Obviously, it is important to consider that these results are correlations, and future studies are needed in order to confirm a direct pro-inflammatory and/or profibrotic role of each fungal species in IBD.

Very little is known about the intestinal mycobiome in complicated-patients with CD and, as far as we are concerned, this is the first study describing a specific fungal signature in intestinal resections from B2-CD and B3-patients with CD. We did not find significant differences in the diversity, richness or dominance indices in complicated-patients with CD when comparing them against control CD samples. When the composition of the intestinal resection-associated mycobiome in patients with complicated CD was compared with that of control CD samples, a significant increase in *Malassezia globose* in B2-CD and a significant reduction in *Candida unclassified* and *Yarrowia lipolytica* in B3-CD samples were found. Moreover, it is important to highlight that in B2-CD samples, *Malassezia globose* and *Malassezia restricta* showed a strong and positive correlation with the expression of *COL1A1* and *COL3A1*. Hence, the increase detected in patients with B2 with CD in *Malassezia globose*, joined with the positive correlations with *COL1A1* and *COL3A1*, suggest that this fungus might contribute to the perpetuation of intestinal inflammation and fibrosis. In line with this, it has been previously reported that *Malassezia* can induce a broad range of pro-inflammatory cytokines including TNF-α, IL-12p40, IL-6 and IL-1β, among others, and even trigger the activation of the NLRP3 inflammasome.^56^ On the other hand, Zeng *et al.* reported alterations in the fecal mycobiome composition associated with clinical phenotypes of patients with CD and an increase in the abundance of the genus *Candida* in non-B1 samples.^57^ The fact that the authors did not distinguish between B2 and B3, together with our observation of a reduction of this genus specifically in B3-CD samples and an increase of *Malassezia globose* in patients with B2 with CD, directly associated *Candida unclassified* and *Malassezia globose* to intestinal stenosis. In line with this, it is important to note that *Candida* increases the severity of murine DSS colitis through the induction of a bacterial dysbiosis and the enhancement of the expression of pro-inflammatory cytokines in the intestine and even systemically in serum.^58^ Moreover, a study reported that an intestinal overgrowth of *Candida albicans* exacerbates bleomycin-induced pulmonary fibrosis in mice.^59^ However, the specific role of these fungi in intestinal inflammation and fibrosis in complicated-CD phenotypes is still a question that needs to be addressed by future studies.

When complicated-CD phenotypes were compared, B2-CD samples exhibited a significant increase in *Dothideomycetes unclassified* in parallel with a significant reduction of *Crytococcus pseudolongus* and *Cladosporium unclassified* in comparison to CD-B3. Moreover, although they can be cause or consequence of the complicated phenotype, the AUC values of *Dothideomycetes unclassified* and *Cladosporium unclassified* strongly suggest that both fungi can discriminate between CD-associated phenotypes and might be directly involved in the pathophysiology of the development of intestinal fibrosis and fistulas, respectively. The lack of any study that describes the role of *Dothideomycetes* in fibrosis raises a new protagonist which needs to be further studied in this CD-complication. On the other hand, though nothing has been demonstrated specifically in fistula, the pathogenic role of *Cladosporium* has been reported in Colitis-Associated Colorectal Cancer.^60^ The fact that these fungal species can discriminate between B2 and B3 phenotype, point them to potential fungal biomarkers. Of course, it is important to note that surgical resections have been obtained when these complicated phenotypes have been stablished. Therefore, future studies are needed in order to analyze whether these fungal species could predict the development of these phenotypes. Indeed, the identification of a fungal signature which could predict the progression of a complicated CD-phenotype would be a milestone in the treatment of patients with CD.

In conclusion, we have characterized for the first time according to our knowledge the intestinal mycobiome in surgical resections from both patients with UC and CD and report statistical differences in the genera *Malassezia* and *Yarrowia*. Several associations between fungal and bacterial species have been identified, thereby reaffirming the direct interaction between these microorganisms. Moreover, positive and significant correlations have been detected between some fungal species and the expression of both pro-inflammatory and profibrotic genes, suggesting a pathogenic role of specific fungal species. Finally, complicated-CD phenotypes exhibit a specific mycobiome, and *Dothideomycetes unclassified* and *Cladosporium unclassified* can be used to discriminate between the 2 phenotypes and should be further studied in order to better understand the development of intestinal fibrosis and fistula.

## Supplementary Material

izaf178_Supplementary_Data

## Data Availability

The data underlying this article are available in the article and in its online supplementary material.

## References

[1] Fan Y , Zhang L, Omidakhsh N, et al Patients with stricturing or penetrating Crohn’s disease phenotypes report high disease burden and treatment needs. Inflamm Bowel Dis. 2023;29:914-922. 35880838 10.1093/ibd/izac162 PMC10233399

[2] Cho CW , You MW, Oh CH, Lee CK, Moon SK. Long-term disease course of Crohn’s disease: changes in disease location, phenotype, activities, and predictive factors. Gut Liver. 2022;16:157-170. 34456186 10.5009/gnl210118 PMC8924800

[3] Vich Vila A , Zhang J, Liu M, Faber KN, Weersma RK. Untargeted fecal metabolomics for the discovery of biomarkers and treatment targets for inflammatory bowel diseases. Gut. 2024;73:1909-1920. 39002973 10.1136/gutjnl-2023-329969 PMC11503092

[4] Cani PD. Human gut microbiome: hopes, threats and promises. Gut. 2018;67:1716-1725. 29934437 10.1136/gutjnl-2018-316723 PMC6109275

[5] Balderramo DC , Romagnoli PA, Granlund AVB, Catalan-Serra I. Fecal fungal microbiota (Mycobiome) study as a potential tool for precision medicine in inflammatory bowel disease. Gut Liver. 2023;17:505-515. 37305948 10.5009/gnl220537 PMC10352062

[6] Jaswal K , Todd OA, Behnsen J. Neglected gut microbiome: interactions of the non-bacterial gut microbiota with enteric pathogens. Gut Microbes. 2023;15:2226916. 37365731 10.1080/19490976.2023.2226916 PMC10305517

[7] Buttar J , Kon E, Lee A, Kaur G, Lunken G. Effect of diet on the gut mycobiome and potential implications in inflammatory bowel disease. Gut Microbes. 2024;16:2399360. 39287010 10.1080/19490976.2024.2399360 PMC11409510

[8] Nash AK , Auchtung TA, Wong MC, et al The gut mycobiome of the Human Microbiome Project healthy cohort. Microbiome. 2017;5:153. 29178920 10.1186/s40168-017-0373-4 PMC5702186

[9] Sokol H , Leducq V, Aschard H, et al Fungal microbiota dysbiosis in IBD. Gut. 2017;66:1039-1048. 26843508 10.1136/gutjnl-2015-310746 PMC5532459

[10] Catalan-Serra I , Thorsvik S, Beisvag V, et al Fungal microbiota composition in inflammatory bowel disease patients: characterization in different phenotypes and correlation with clinical activity and disease course. Inflamm Bowel Dis. 2024;30:1164-1177. 38103028 10.1093/ibd/izad289 PMC11219482

[11] Chehoud C , Albenberg LG, Judge C, et al Fungal signature in the gut microbiota of pediatric patients with inflammatory bowel disease. Inflamm Bowel Dis. 2015;21:1948-1956. 26083617 10.1097/MIB.0000000000000454 PMC4509842

[12] Hoarau G , Mukherjee PK, Gower-Rousseau C, et al Bacteriome and mycobiome interactions underscore microbial dysbiosis in familial Crohn’s disease. mBio. 2016;7:10.1128/mBio.01250-16 PMC5030358 27651359

[13] Standaert-Vitse A , Sendid B, Joossens M, et al Candida albicans colonization and ASCA in familial Crohn’s disease. Am J Gastroenterol. 2009;104:1745-1753. 19471251 10.1038/ajg.2009.225

[14] Ventin-Holmberg R , Eberl A, Saqib S, et al Bacterial and fungal profiles as markers of infliximab drug response in inflammatory bowel disease. J Crohns Colitis. 2021;15:1019-1031. 33300552 10.1093/ecco-jcc/jjaa252

[15] Leonardi I , Paramsothy S, Doron I, et al Fungal trans-kingdom dynamics linked to responsiveness to fecal microbiota transplantation (FMT) therapy in ulcerative colitis. Cell Host Microbe. 2020;27:823-829.e3. e823. 32298656 10.1016/j.chom.2020.03.006 PMC8647676

[16] Bauset C , Carda-Dieguez M, Cejudo-Garces A, et al A disturbed metabolite-GPCR axis is associated with microbial dysbiosis in IBD patients: Potential role of GPR109A in macrophages. Biochim Biophys Acta Mol Basis Dis. 2024;1870:167489. 39233260 10.1016/j.bbadis.2024.167489

[17] Boix-Amoros A , Martinez-Costa C, Querol A, Collado MC, Mira A. Multiple approaches detect the presence of fungi in human breastmilk samples from healthy mothers. Sci Rep. 2017;7:13016. 29026146 10.1038/s41598-017-13270-x PMC5638952

[18] Gardes M , Bruns TD. ITS primers with enhanced specificity for basidiomycetes—application to the identification of mycorrhizae and rusts. Mol Ecol. 1993;2:113-118. 8180733 10.1111/j.1365-294x.1993.tb00005.x

[19] Callahan BJ , McMurdie PJ, Rosen MJ, Han AW, Johnson AJ, Holmes SP. DADA2: high-resolution sample inference from Illumina amplicon data. Nat Methods. 2016;13:581-583. 27214047 10.1038/nmeth.3869 PMC4927377

[20] Quast C , Pruesse E, Yilmaz P, et al The SILVA ribosomal RNA gene database project: improved data processing and web-based tools. Nucleic Acids Res. 2013;41:D590-596. 23193283 10.1093/nar/gks1219 PMC3531112

[21] de la Cuesta-Zuluaga J , Kelley ST, Chen Y, et al Age- and sex-­dependent patterns of gut microbial diversity in human adults. mSystems. 2019;4. 10.1128/msystems.00261-19 PMC6517691 31098397

[22] Rustgi SD , Kayal M, Shah SC. Sex-based differences in inflammatory bowel diseases: a review. Therap Adv Gastroenterol. 2020;13:1756284820915043. 10.1177/1756284820915043 PMC7236567 32523620

[23] Fang L , Peng H, Tan Z, Deng N, Peng X. The role of gut microbiota on intestinal fibrosis in inflammatory bowel disease and traditional Chinese medicine intervention. J Inflamm Res. 2025;18:5951-5967. 40357383 10.2147/JIR.S504827 PMC12067688

[24] Huang H , Wang Q, Yang Y, Zhong W, He F, Li J. The mycobiome as integral part of the gut microbiome: crucial role of symbiotic fungi in health and disease. Gut Microbes. 2024;16:2440111. 39676474 10.1080/19490976.2024.2440111 PMC11651280

[25] Mar Rodriguez M , Perez D, Javier Chaves F, et al Obesity changes the human gut mycobiome. Sci Rep. 2015;5:14600. 26455903 10.1038/srep14600 PMC4600977

[26] Lof M , Janus MM, Krom BP. Metabolic interactions between bacteria and fungi in commensal oral biofilms. J Fungi (Basel). 2017;3:40. 29371557 10.3390/jof3030040 PMC5715944

[27] Iliev ID , Funari VA, Taylor KD, et al Interactions between commensal fungi and the C-type lectin receptor Dectin-1 influence colitis. Science. 2012;336:1314-1317. 22674328 10.1126/science.1221789 PMC3432565

[28] Hsu C , Ghannoum M, Cominelli F, Martino LD. Mycobiome and inflammatory bowel disease: role in disease pathogenesis, current approaches and novel nutritional-based therapies. Inflamm Bowel Dis. 2023;29:470-479. 35851921 10.1093/ibd/izac156 PMC9977251

[29] Underhill DM , Braun J. Fungal microbiome in inflammatory bowel disease: a critical assessment. J Clin Invest. 2022;132:e155786. 10.1172/JCI155786 PMC8884899 35229726

[30] Krawczyk A , Salamon D, Kowalska-Duplaga K, et al Changes in the gut mycobiome in pediatric patients in relation to the clinical activity of Crohn’s disease. World J Gastroenterol. 2023;29:2172-2187. 37122605 10.3748/wjg.v29.i14.2172 PMC10130967

[31] Qiu X , Zhao X, Cui X, et al Characterization of fungal and bacterial dysbiosis in young adult Chinese patients with Crohn’s disease. Therap Adv Gastroenterol. 2020;13:1756284820971202. 10.1177/1756284820971202 PMC7672770 33240394

[32] Li Q , Wang C, Tang C, He Q, Li N, Li J. Dysbiosis of gut fungal microbiota is associated with mucosal inflammation in Crohn’s disease. J Clin Gastroenterol. 2014;48:513-523. 24275714 10.1097/MCG.0000000000000035 PMC4059552

[33] Liguori G , Lamas B, Richard ML, et al Fungal dysbiosis in mucosa-associated microbiota of Crohn’s disease patients. J Crohns Colitis. 2016;10:296-305. 26574491 10.1093/ecco-jcc/jjv209 PMC4957473

[34] Qiu X , Ma J, Jiao C, et al Alterations in the mucosa-associated fungal microbiota in patients with ulcerative colitis. Oncotarget. 2017;8:107577-107588. 29296188 10.18632/oncotarget.22534 PMC5746090

[35] Scanu M , Toto F, Petito V, et al An integrative multi-omic analysis defines gut microbiota, mycobiota, and metabolic fingerprints in ulcerative colitis patients. Front Cell Infect Microbiol. 2024;14:1366192. 38779566 10.3389/fcimb.2024.1366192 PMC11109417

[36] Jangi S , Moyer J, Sandlow S, et al Microbial butyrate capacity is reduced in inflamed mucosa in patients with ulcerative colitis. Sci Rep. 2024;14:3479. 38347087 10.1038/s41598-024-54257-9 PMC10861456

[37] Jangi S , Hsia K, Zhao N, et al Dynamics of the gut mycobiome in patients with ulcerative colitis. Clin Gastroenterol Hepatol. 2024;22:821-830.e7.e827. 37802272 10.1016/j.cgh.2023.09.023 PMC10960711

[38] Wetzel S , Muller A, Kohnert E, et al Longitudinal dynamics of gut bacteriome and mycobiome interactions pre- and post-visceral surgery in Crohn’s disease. Front Cell Infect Microbiol. 2023;13:1275405. 38287975 10.3389/fcimb.2023.1275405 PMC10822897

[39] Limon JJ , Tang J, Li D, et al Malassezia is associated with Crohn’s disease and exacerbates colitis in mouse models. Cell Host Microbe. 2019;25:377-388 e376. 30850233 10.1016/j.chom.2019.01.007 PMC6417942

[40] Olaisen M , Richard ML, Beisvag V, et al The ileal fungal microbiota is altered in Crohn’s disease and is associated with the disease course. Front Med (Lausanne). 2022;9:868812. 36237548 10.3389/fmed.2022.868812 PMC9551188

[41] Ha CWY , Martin A, Sepich-Poore GD, et al Translocation of viable gut microbiota to mesenteric adipose drives formation of creeping fat in humans. Cell. 2020;183:666-683 e617. 32991841 10.1016/j.cell.2020.09.009 PMC7521382

[42] Gouba N , Drancourt M. Digestive tract mycobiota: a source of infection. Med Mal Infect. 2015;45:9-16. 25684583 10.1016/j.medmal.2015.01.007

[43] Kazemian N , Ramezankhani M, Sehgal A, et al The trans-kingdom battle between donor and recipient gut microbiome influences IL-6 microbiota transplantation outcome. Sci Rep. 2020;10:18349. 33110112 10.1038/s41598-020-75162-x PMC7591866

[44] Tullio V. Probiotic Yeasts: A Developing Reality? J Fungi (Basel). 2024;10:489. 39057374 10.3390/jof10070489 PMC11277836

[45] Li XV , Leonardi I, Putzel GG, et al Immune regulation by fungal strain diversity in inflammatory bowel disease. Nature. 2022;603:672-678. 35296857 10.1038/s41586-022-04502-w PMC9166917

[46] Kaeuffer C , Baldacini M, Ruge T, et al Fungal infections caused by Kazachstania spp., Strasbourg, France, 2007–2020. Emerg Infect Dis. 2022;28:29-34. 34932452 10.3201/eid2801.211543 PMC8714217

[47] Liu MM , Zhu HH, Bai J, et al Breast cancer colonization by Malassezia globosa accelerates tumor growth. mBio. 2024;15:e0199324. 39235230 10.1128/mbio.01993-24 PMC11481877

[48] Xie Z , Canalda-Baltrons A, d‘Enfert C, Manichanh C. Shotgun metagenomics reveals interkingdom association between intestinal bacteria and fungi involving competition for nutrients. Microbiome. 2023;11:275. 38098063 10.1186/s40168-023-01693-w PMC10720197

[49] Liu HY , Li S, Ogamune KJ, et al Fungi in the gut microbiota: interactions, homeostasis, and host physiology. Microorganisms. 2025;13:70. 39858841 10.3390/microorganisms13010070 PMC11767893

[50] Loster JE , Wieczorek A, Loster BW. Correlation between age and gender in Candida species infections of complete denture wearers: a retrospective analysis. Clin Interv Aging. 2016;11:1707-1714. 27920509 10.2147/CIA.S116658 PMC5123722

[51] Firrman J , Deyaert S, Mahalak KK, et al The bifidogenic effect of 2′fucosyllactose is driven by age-specific bifidobacterium species, demonstrating age as an important factor for gut microbiome targeted precision medicine. Nutrients. 2024;17. 10.3390/nu17010151 PMC11723031 39796584

[52] Strati F , Di Paola M, Stefanini I, et al Age and gender affect the composition of fungal population of the human gastrointestinal tract. Front Microbiol. 2016;7:1227. 27536299 10.3389/fmicb.2016.01227 PMC4971113

[53] Ma X , Hu J, Wang C, et al Innate and mild Th17 cutaneous immune responses elicited by subcutaneous infection of immunocompetent mice with Cladosporium cladosporioides. Microb Pathog. 2022;163:105384. 34974124 10.1016/j.micpath.2021.105384

[54] Ma X , Hu J, Yu Y, et al Assessment of the pulmonary adaptive immune response to Cladosporium cladosporioides infection using an experimental mouse model. Sci Rep. 2021;11:909. 33441700 10.1038/s41598-020-79642-y PMC7806624

[55] Jain U , Ver Heul AM, Xiong S, et al Debaryomyces is enriched in Crohn’s disease intestinal tissue and impairs healing in mice. Science. 2021;371:1154-1159. 33707263 10.1126/science.abd0919 PMC10114606

[56] Wolf AJ , Limon JJ, Nguyen C, Prince A, Castro A, Underhill DM. Malassezia spp. induce inflammatory cytokines and activate NLRP3 inflammasomes in phagocytes. J Leukoc Biol. 2021;109:161-172. 32941658 10.1002/JLB.2MA0820-259R PMC7902306

[57] Zeng L , Feng Z, Zhuo M, et al Fecal fungal microbiota alterations associated with clinical phenotypes in Crohn’s disease in southwest China. PeerJ. 2022;10:e14260. 36275466 10.7717/peerj.14260 PMC9586077

[58] Panpetch W , Hiengrach P, Nilgate S, et al Additional Candida albicans administration enhances the severity of dextran sulfate solution induced colitis mouse model through leaky gut-­enhanced systemic inflammation and gut-dysbiosis but attenuated by ­Lactobacillus rhamnosus L34. Gut Microbes. 2020;11:465-480. 31530137 10.1080/19490976.2019.1662712 PMC7527076

[59] Yamada T , Nakashima T, Masuda T, et al Intestinal overgrowth of Candida albicans exacerbates bleomycin-induced pulmonary fibrosis in mice with dysbiosis. J Pathol. 2023;261:227-237. 37565293 10.1002/path.6169

[60] Wu M , Li J, An Y, et al Chitooligosaccharides prevents the development of colitis-associated colorectal cancer by modulating the intestinal microbiota and mycobiota. Front Microbiol. 2019;10:2101. 31620100 10.3389/fmicb.2019.02101 PMC6759605

