# Supplementary material for: Characterization of Intestinal Mycobiome in Surgical Resections from Inflammatory Bowel Disease Patients: A Deeper Analysis in Complicated Crohn’s Disease Phenotypes
Source: Inflamm Bowel Dis. 2025 Oct 30;31(12):3256–70. doi: 10.1093/ibd/izaf178 (PMC12688065; doi:10.1093/ibd/izaf178)
Supplement: izaf178_Supplementary_Data [file izaf178_supplementary_data.zip › Legend Supplementary Figure 1.docx]

**Supplementary Figure 1. Donor-specific breakdown of fungal relative abundance.** A) The data of fungal relative abundance at both genus and species levels are shown broken down by individual patient in control-UC, UC, control-CD and CD groups. B) The data of fungal relative abundance at both genus and species levels are shown broken down by individual patient in B2-CD and B3-CD groups.
