# Supplementary material for: Characterization of Intestinal Mycobiome in Surgical Resections from Inflammatory Bowel Disease Patients: A Deeper Analysis in Complicated Crohn’s Disease Phenotypes
Source: Inflamm Bowel Dis. 2025 Oct 30;31(12):3256–70. doi: 10.1093/ibd/izaf178 (PMC12688065; doi:10.1093/ibd/izaf178)
Supplement: izaf178_Supplementary_Data [file izaf178_supplementary_data.zip › Supplementary Table 1 Manuscript IBD.docx]

Supplementary Table 1. Receiver-operating characteristic (ROC) curves of the most abundant fungal species detected in B2-CD and B3-CD intestinal surgical resections.

| Fungal species | AUC | p value | 95% CI |
| --- | --- | --- | --- |
| *Saccharomyces kudriavzevii* | 0,6515 | 0,1775 | 0,4430-0,8600 |
| *Penicillium unclassified* | 0,6667 | 0,1380 | 0,4543-0,8791 |
| *Malassezia restricta* | 0,5808 | 0,4721 | 0,3709-0,7907 |
| *Saccharomyces paradoxus* | 0,6061 | 0,3452 | 0,3951-0,8170 |
| *Candida albicans* | 0,5909 | 0,4185 | 0,3618-0,8200 |
| ***Dothideomycetes unclassified*** | **0,8232** | **0,0040** | **0,6682-0,9783** |
| *Yarrowia lipolytica* | 0,5556 | 0,6210 | 0,3413-0,7698 |
| ***Cladosporium unclassified*** | **0,7374** | **0,0346** | **0,5225-0,9523** |
| *Malassezia globose* | 0,6919 | 0,0876 | 0,4832-0,9006 |
| *Debaryomyces unclassified* | 0,5859 | 0,4448 | 0,3743-0,7974 |
| *Trichoderma unclassified* | 0,5253 | 0,8222 | 0,3035-0,7470 |
| *Candida unclassified* | 0,6465 | 0,1924 | 0,4434-0,8495 |
| *Kazachstania unclassified* | 0,5455 | 0,6858 | 0,3249-0,7660 |
