# Supplementary figures and images for: Characterization of Intestinal Mycobiome in Surgical Resections from Inflammatory Bowel Disease Patients: A Deeper Analysis in Complicated Crohn’s Disease Phenotypes
Source: Inflamm Bowel Dis. 2025 Oct 30;31(12):3256–70. doi: 10.1093/ibd/izaf178 (PMC12688065; doi:10.1093/ibd/izaf178)

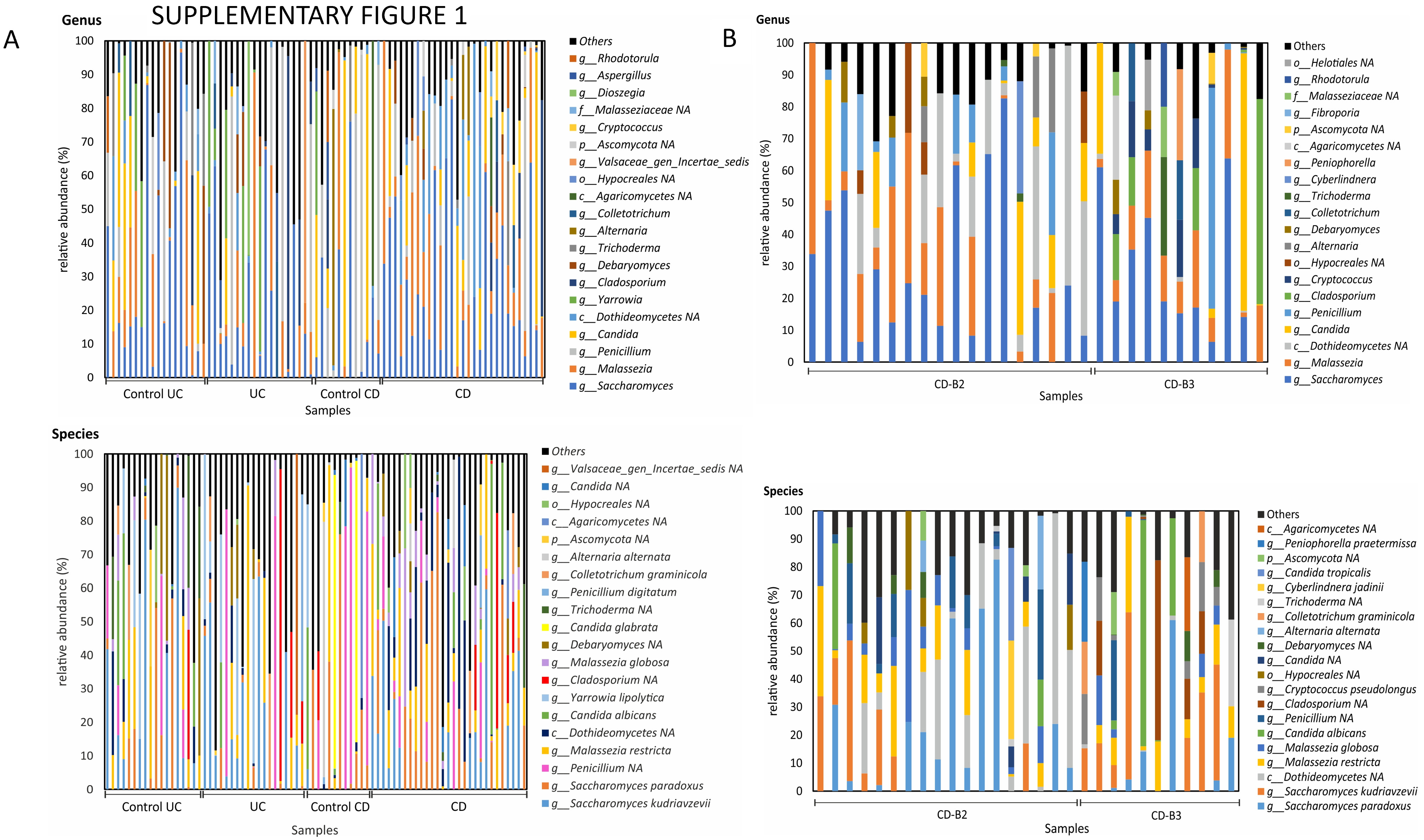

Supplement: izaf178_Supplementary_Data [file izaf178_supplementary_data.zip › Figure S1.tiff]
